# Use of Home Care Services Reduces Caregiving-Related Strain in Long-Distance Caregivers

**DOI:** 10.1093/geroni/igaa057.1896

**Published:** 2020-12-16

**Authors:** Francesca Falzarano, Verena Cimarolli, Kathrin Boerner, Amy Horowitz, Karen Siedlecki

**Affiliations:** 1 Weill Cornell Medicine, New York, New York, United States; 2 LeadingAge, Washington, District of Columbia, United States; 3 University of Massachusetts Boston, Boston, Massachusetts, United States; 4 Fordham University, New York, New York, United States

## Abstract

Long-distance caregivers (LDCs) comprise a growing yet relatively understudied segment of the caregiving population. The current study (N=166) investigated the mediating role of home care hours on the association between primary caregiving stressors (care recipient [CR] levels of cognitive and functional impairment) and LDCs’ perceived strain (perceived interference of caregiving with other family responsibilities and work). Results from path analyses showed that home care hours fully mediated the negative effect of CRs’ functional impairment on family interference and the negative effect of CRs’ cognitive impairment on work interference. Further, the negative effect of CRs’ cognitive impairment on family interference was partially mediated by home care hours. Thus, a mediating role of home care service hours in the associations between objective stressors and LDC strain was established for both family and work domains. Findings highlight the potential of home care services in alleviating strain in LDCs of community-dwelling CRs.

